# High-Efficiency Purification and Morphology Regulation of CaSO_4_·2H_2_O Crystals from Phosphogypsum

**DOI:** 10.3390/molecules29163910

**Published:** 2024-08-19

**Authors:** Yang Lei, Yong-Ji Gong, Min He, Liangqun Li, Jun Qin, Yufei Liu

**Affiliations:** 1 College of Materials and Metallurgy, Guizhou University, Guiyang 550025, China; leiyang3735@126.com (Y.L.); yjgong159@163.com (Y.-J.G.); 2 Key Laboratory of Chemistry for Natural Products of Guizhou Province and Chinese Academy of Sciences, Guiyang 550014, China; liliangqun2010@163.com; 3 Key Laboratory of Karst Georesources and Environment, Ministry of Education, College of Resources and Environmental Engineering, Guizhou University, Guiyang 550025, China; qi_njun@163.com; 4 National Engineering Research Center for Compounding and Modification of Polymeric Materials, Guiyang 550014, China

**Keywords:** phosphogypsum, morphology regulation, Cu^2+^ loading

## Abstract

Phosphogypsum is a solid waste with great environmental stockpile pressure produced by the wet production of phosphoric acid. Although there are various methods to purify and utilize phosphogypsum, the means for environmentally friendly, low energy consumption, and high value-added utilization still need to be further explored. Here, CaSO_4_·2H_2_O crystal was directly purified and regulated from phosphogypsum by using the anti-solvent method. The antisolvent can be adsorbed in the *c*-axis direction of the crystal and further inhibit the growth rate in this direction, resulting in a change in the morphology of the crystal. By adjusting the polarity and chain length of the anti-solvent, the morphology of CaSO_4_·2H_2_O crystal can change from butterfly-like flake crystals to hexagonal prism-like crystals. When n-propanol with long chain was used as the anti-solvent, the morphology of the CaSO_4_·2H_2_O crystal showed a hexagonal prism with a specific surface area of 19.98 m^2^/g and a Cu^2+^ loading efficiency of 52.67%. The encouraging results open up new possibilities for the application of phosphogypsum.

## 1. Introduction

Phosphogypsum is a waste from the phosphorus chemical industry. The accumulation of phosphogypsum leads to the release of harmful substances that can pollute the air, soil, rivers, or groundwater [1], accumulate in the food chain through enrichment, and cause public health problems [2]. Although the main component of phosphogypsum is CaSO_4_·2H_2_O and other impurities, the impurities in phosphogypsum greatly limit its application. The current impurity removal methods include: (1) water washing can remove the acidic and soluble impurities of phosphogypsum, but it is difficult to remove insoluble impurities, and the water resource consumption is serious [3]. (2) Flotation can only remove organics [4]. (3) The calcination method can only remove eutectic phosphorus and organic matter, but it consumes high energy [5]. In addition, acid leaching is a relatively effective purification technology; the acid is selective to impurities, and it is difficult to use a single acid to effectively purify phosphogypsum [6,7,8]. The quicklime neutralization method also has the problem of difficulty in removing insoluble impurities [4]. Although the purification effect is good, the process is very complicated. The anti-solvent method [9] has a simple treatment process and mild reaction conditions. This method is environmentally friendly for extracting CaSO_4_·2H_2_O from phosphogypsum and can efficiently precipitate CaSO_4_·2H_2_O crystals from water because the anti-solvent makes CaSO_4_·2H_2_O solubility to produce a dramatic change [10]. However, the morphological regulation of CaSO_4_·2H_2_O crystals has been difficult to realize due to the fast precipitation speed and elusive precipitation mechanism of the anti-solvent method.

The morphology of CaSO_4_·2H_2_O also has a great influence on the utilization of the materials engineer [11,12,13]. In previous research, the morphology regulation of CaSO_4_·2H_2_O is mainly regulated by the addition of crystal form regulators. The common crystal form regulators include polymer additives [14], metal ions [15,16,17], calcium ion complexing agents [18], hexadecyl pyridine chloride [19], amino acids [20], and oxalic acid [21]. The addition of crystal form modifiers is mostly used in the morphology adjustment of pure CaSO_4_·2H_2_O, which is limited by the complex multi-component of phosphogypsum. The addition of a crystal form agent in phosphogypsum will also have more uncontrollable factors, such as inactivation in the acidic environment of phosphogypsum or adsorption by other components, which result in difficult purifying CaSO_4_·2H_2_O. In addition, the morphology regulation of CaSO_4_·2H_2_O crystal mainly focused on the third group of CaSO_4_·2H_2_O, the influence of solvent on the growth mechanism was mostly ignored in the research process [22].

In this work, CaSO_4_·2H_2_O was directly purified from phosphogypsum by anti-solvent (method: ethanol and n-propanol) to regulate the morphology of CaSO_4_·2H_2_O crystals. The obtained butterfly-shaped, hexagonal prism-shaped, and mixed-shaped three-dimensional CaSO_4_·2H_2_O crystals have different morphologies and scales. The method has the advantages of excellent ability of morphology regulation, high purity efficiency, simple experimental operation, mild reaction conditioning, being environmentally friendly, and low energy consumption. Further studies on the mechanism of regulation showed that the anti-solvent adsorbed on the *c*-axis direction of the CaSO_4_·2H_2_O crystal through the van der Waals interaction, inhibiting the growth rate of the CaSO_4_·2H_2_O crystal in the *c*-axis direction to effectively regulate the morphology. Generally, the adsorption capacity of the CaSO_4_·2H_2_O crystal on the anti-solvent will increase with an increase in the polarity of the anti-solvent. The inhibitory effect of the anti-solvent on the CaSO_4_·2H_2_O crystal will increase with the addition of the anti-solvent’s chain length. Herein, the morphology of CaSO_4_·2H_2_O crystal can be controlled effectively by adjusting the polarity and chain length of the antisolvent molecules. When n-propanol was used as the anti-solvent, the morphology of the CaSO_4_·2H_2_O crystal showed a hexagonal prism with a specific surface area of 19.98 m^2^/g and a Cu^2+^ loading efficiency of 52.67%. In addition, the purified CaSO_4_·2H_2_O crystal from phosphogypsum has excellent biocompatibility and great application potential in the field of biomedicine, which provides new ideas for the application of phosphogypsum.

## 2. Results and Discussion

**Figure 1:**
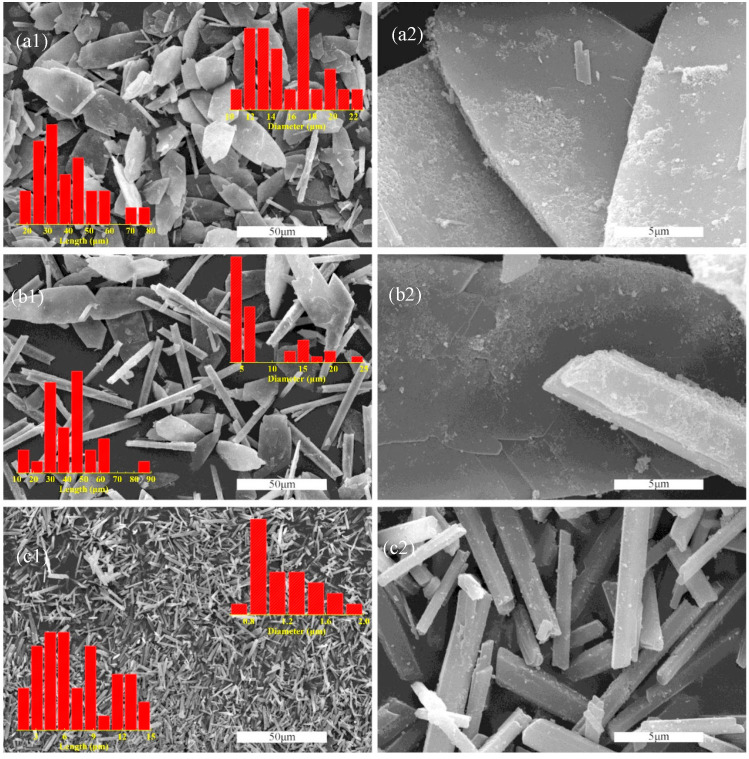
SEM images of (**a**) Product A, (**b**) Product B, and (**c**) Product C were acquired at 3:10 mixing ratios. Images (**a2**,**b2**,**c2**) are a 10× magnification of images (**a1**,**b1**,**c1**).

As shown in Supplementary Figure S1a, the phosphogypsum after natural drying is a gray solid powder. It can be seen from a SEM image (Figure S1b, Supplementary Materials) that the particles of phosphogypsum show multi-scale distribution, including quadrilateral block crystals ranging from a dozen microns to tens of microns, short columnar crystals with a few microns in diameter and tens of microns in length, and regular particles with a large difference in scale. The PXRD pattern of phosphogypsum demonstrates that the main components of phosphogypsum are calcium sulfate dihydrate and silica (Figure S1c, Supplementary Materials). In addition, the XPS shows that the constituent elements of phosphogypsum, which include P, F, and Si in addition to S, O, and Ca (Figure S1d, Supplementary Materials). The utilization processes of phosphogypsum are generally complex and high energy consumptive. To solve the problems, an anti-solvent method with simple operation, environmental protection, and high product purity is selected to depurate the phosphogypsum and modulate the microstructure. The morphology of the product was successfully controlled by changing the chain length of the anti-solvent (the mixing ratio of methanol, ethanol, and n-propanol to solution is 3:10, respectively). The morphologies of the obtained products are characterized by SEM, as shown in Figure 1. With the increase in anti-solvent chain length, the morphology of the product changes from butterfly-like flake crystal to hexagonal prism-like crystal. The morphology of Product A (methanol is used as an anti-solvent) is almost always butterfly-shaped flake crystals with a width of 10~20 μm and a length of 20~80 μm. Product B (ethanol is used as an anti-solvent) consists of two morphological crystals; one is a butterfly-shaped flake crystal with a width distribution of 10~25 μm, and the other is a hexagonal prism-shaped crystal with a width distribution of t 3~7 μm and a length distribution of 10~70 μm. The morphology of Product C (n-propanol is used as an anti-solvent) is not only almost a hexagonal prismatic crystal but also the width and length are reduced to 0.6~2 μm and 1~15 μm, respectively. The SEM micrographs of Products C1, C2, and C3, acquired at varying mixing ratios of anti-solvent (2:10, 4:10, and 5:10), reveal that the size of the products is influenced by the mixing ratio, while the geometric morphology remains consistent across all ratios (Figure S2, Supplementary Materials).

**Figure 2:**
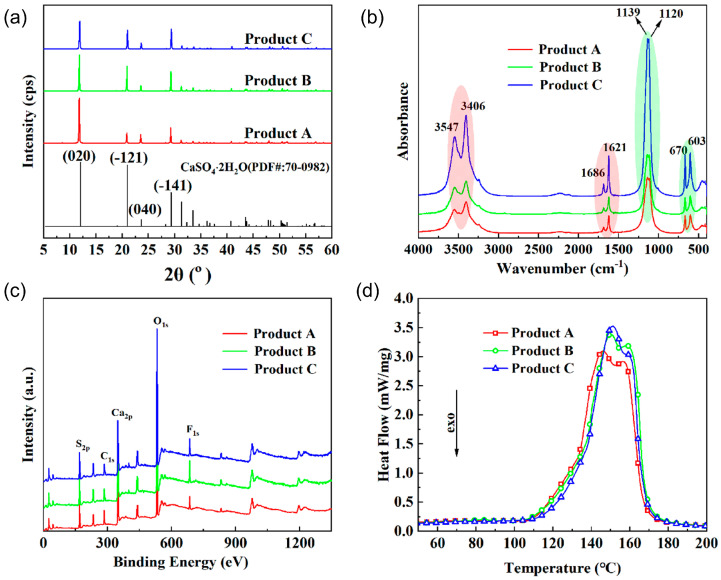
The analysis of product composition and purity. (**a**) PXRD curves. (**b**) FTIR spectra. (**c**) XPS spectra. (**d**) DSC curves.

The chain length change of the anti-solvent can regulate the morphology and micro-size of the corresponding product, which enhances the application potential of these products. To identify the safety and reliability of the product, the components and purity of these products are measured by XRD, FTIR, XPS, and DSC. The diffraction patterns of the products match the standard characteristic peaks of the (020), (-121), (040), and (-141) crystal faces of the CaSO_4_·2H_2_O, as shown in Figure 2a and Figure S3 (Supplementary Materials). This congruence confirms that the products are composed of CaSO_4_·2H_2_O and demonstrates the phase purity for Products A, B, and C. Figure 2b shows multiple vibrational absorption bands of these products; these vibration peaks demonstrate that the main component of the product is CaSO_4_·2H_2_O. In gypsum molecules, the geometry of SO_4_ deviates from the ideal tetrahedral configuration (Td symmetry group) to the lower molecular symmetry C_2_ [23,24]. Therefore, a weak band at 1005 cm^−1^ is observed in the FTIR spectrum of gypsum, which corresponds to vibrational absorption bands (S–O symmetric stretch) of the SO_4_ tetrahedron in gypsum. This symmetric stretching mode is observed due to the lower symmetry of sulfate-ion in gypsum and is otherwise IR inactive in the free sulfate-ion [25]. The polarity of n-propanol is quite different from that of water molecules, and it has the greatest influence on the structure of SO_4_ during the crystallization process, and the ν_1_ of Product C is the most obvious. In addition, there are four more distinctive peaks here. The peaks at 1138 cm^−1^ and 1120 cm^−1^ are assigned to the splitting of the triple-degenerate S–O asymmetric stretching vibration in the free sulfate ion. The peaks at 669 cm^−1^ and 604 cm^−1^ are assigned to antisymmetric bending vibration that is generated by splitting into double peaks for the triple degeneracy of sulfate tetrahedra in gypsum [25]. The peak at 3546 cm^−1^ and 3405 cm^−1^ corresponded to vibrational absorption bands of hydroxyl (–OH), and the peak at 1685 cm^−1^ and 1621 cm^−1^ corresponded to bending vibration peaks of –OH in water. The XPS is used to further confirm the constituent elements of these products. Figure 2c shows the constituent elements of these products (Ca, S, O, C, and F elements), where the Ca, S, and O elements correspond to the CaSO_4_·2H_2_O. in the product, and the F element is soluble F for precipitating together with the product during the crystallization process of the product in phosphogypsum (the C element comes from the test background). By integrating the correlative peak areas of each element, the approximate atomic percentage contents of the three main elements Ca, S, and O in the phosphogypsum product were obtained (Table S1, Supplementary Materials). The proportion of Ca, S, and O elements is close to that of 1:1:4, indicating that three products are CaSO_4_·2H_2_O. In addition, the endothermic change during the heating process of the product is also used to indirectly corroborate the composition of the product. As shown in Figure 2d, the DSC curve occurs two endothermic peaks overlapped together in the temperature range from 100 °C to 160 °C, which correspond to the two processes of the dehydration that the CaSO_4_·2H_2_O first loses 3/2H_2_O to become CaSO_4_·1/2H_2_O (Equation (1)) and then loses 1/2H_2_O to transform into γ-CaSO_4_ (Equation (2)) [25], which is consistent with the reported literature. Therefore, we believe that the main component of the product is CaSO_4_·2H_2_O, and the change in the anti-solvent chain length will not affect the composition of the product.

(1) $$\begin{array}{c} CaSO_{4}\cdot 2H_{2}O=CaSO_{4}\cdot 1/2H_{2}O+3/2H_{2}O \end{array}$$

(2) $$\begin{array}{c} CaSO_{4}\cdot 1/2H_{2}O=CaSO_{4}+1/2H_{2}O \end{array}$$

**Figure 3:**
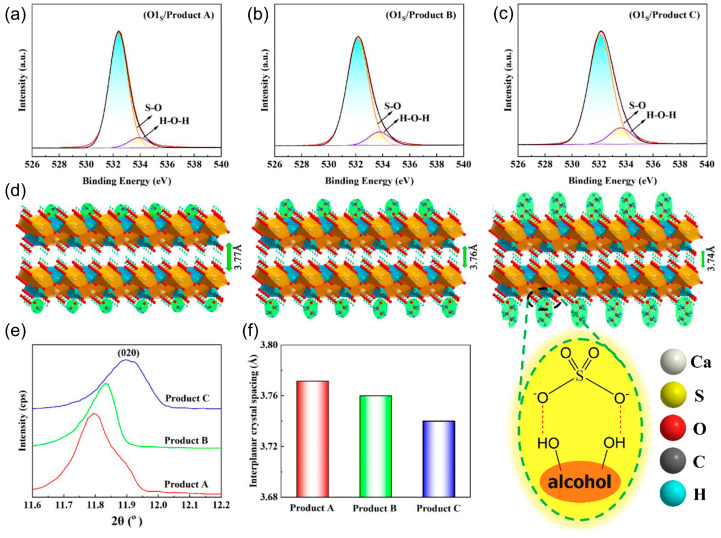
Influence of antisolvent polarity on product structure: (**a**) XPS spectra of O1_S_ of Product A, (**b**) Product B and (**c**) Product C. (**d**) Schematic diagram of the effect of antisolvent on interplanar spacing for (020) crystal plane. (**e**) PXRD curve of the product of (020) crystal plane and (**f**) product of (020) interplanar spacing.

To further understand the mechanism of morphology regulation of CaSO_4_·2H_2_O, the XPS energy spectrum of the O element of these products is used to further investigate the impact of anti-solvent on the morphology of CaSO_4_·2H_2_O. Methanol, ethanol, and n-propanol all contain hydroxyl groups, and the change in the length of the carbon chain connected to the hydroxyl group changes the polarity of the molecule. The polarity of anti-solvent molecules decreases as the length of the carbon chain increases (methanol, 6.6 > ethanol, 4.3 > n-propanol, 4.0) [26]. Figure 3a–c show two kinds of peaks of the O element (S–O and H–O–H) after peak fitting. The S–O and H–O–H peaks are the S–O and H_2_O in CaSO_4_·2H_2_O, respectively. The positions of the characteristic peaks of the oxygen elements of each product are counted in Table S2 (Supplementary Materials). It can be seen that there are differences in the positions of the S–O characteristic peaks of the products obtained under the action of different anti-solvents. The reason for this result is that the anti-solvent will selectively adsorb on the crystal surface during the crystallization process [27]. As shown in Figure 3d, the alcoholic hydroxyl group of the anti-solvent will form a hydrogen bond with the oxygen atom of the sulfate radical in CaSO_4_·2H_2_O, which will be adsorbed in a specific direction of the crystal. In addition, the PXRD peak of the product’s (020) crystal plane is selected to further confirm the impact of the anti-solvent on the crystal growth of CaSO_4_·2H_2_O, as shown in Figure 3e. According to the Bragg equation, the (020) interplanar spacing is calculated and shown in Figure 3f. This result indicates that the interplanar spacing of these products decreases from 3.77 Å to 3.74 Å as the polarity of the anti-solvent decreases because the anti-solvent of the lower polarity will decrease the van der Waals interaction between the anti-solvent and the CaSO_4_·2H_2_O. 

**Figure 4:**
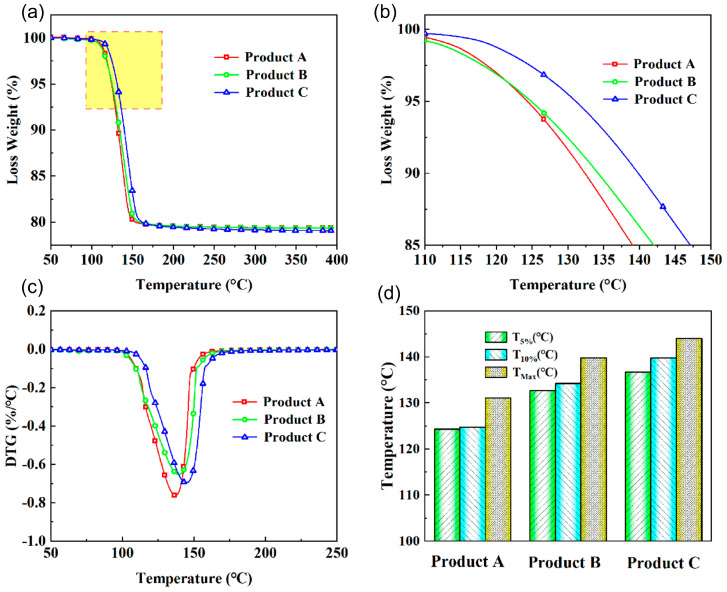
Product thermal stability characterization. (**a**) TG curves of product. (**b**) Enlargement of the yellow square area in Figure 4a. (**c**) DTG curves of product and (**d**) T_5%_ (°C), T_10%_ (°C), and T_Max_ (°C) of product.

Changes in the structure of the product will affect their thermal stability. To further investigate the impact of interplanar spacing on thermal stability, the TG experiment is used to characterize the thermal stability of these products and indirectly prove the change in the product structure. The thermogravimetric curve of these products is shown in Figure 4a. The thermogravimetric curve of these products shows a single weight loss peak, and the temperature range of thermogravimetric loss is 100~160 °C, which is consistent with the DTG results (Figure 4b,c). Furthermore, the maximum weight loss of Products A, B, and C are 20.63%, 20.63%, and 20.93%, respectively, which indicates the component of these products is CaSO_4_·2H_2_O and the loss is the crystal water in CaSO_4_·2H_2_O. However, the thermal stability of these products is slightly different (Figure 4b). The T_5%_, T_10%,_ and T_Max_ of these products are calculated and plotted in Figure 4d (T_5%_, T_10%_, and T_Max_ represent the corresponding temperature at the weight loss of 5%, 10%, and maximum). It can be seen from the figure that T_5%_, T_10%_, and T_Max_ increase with the decrease of the interplanar spacing of the product. Product A with the largest interplanar spacing has T_5%_, T_10%_, and T_Max_ of 124.35 °C, 124.8 °C, and 131.12 °C, respectively, and the T_5%_, T_10%_, and T_Max_ of Product C with the smallest interplanar spacing are 136.74 °C, 139.79 °C, and 144.04 °C, respectively. The reason for this result is the reduction of the interplanar spacing of the product, which will enhance the confinement effect of the product on the water molecules. The change in the thermal stability of the product supports the change of the product structure from the side. 

The above analysis indicates that the polarity of the anti-solvent clearly affects the crystal growth, structure, and thermal stability of the CaSO_4_·2H_2_O. To clearly understand the growth process of CaSO_4_·2H_2_O, the growth direction and rate of the crystal are discussed as two important factors. The CaSO_4_·2H_2_O crystal belongs to the monoclinic system and has three growth directions (*a*-, *b*-, and *c*-axis), of which the *a*-axis direction is enriched with Ca elements and oxygen enrichment in the *c*-axis direction.

**Figure 5:**
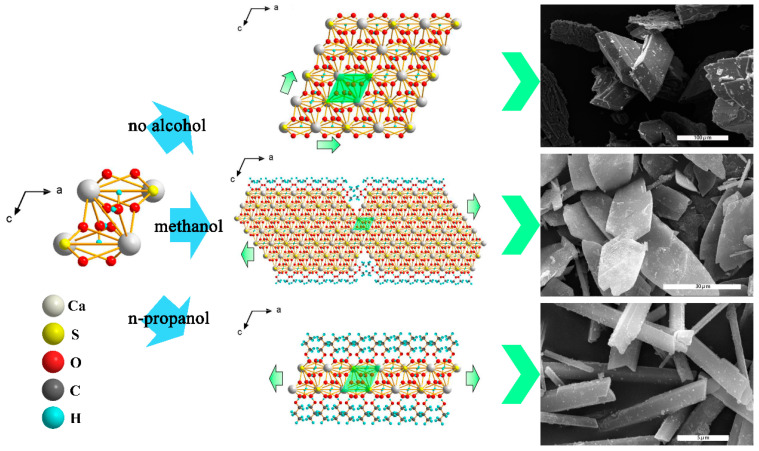
A schematic diagram of the effect of anti-solvent on product morphology.

It can be seen from Figure 5 that the crystal growth of CaSO_4_·2H_2_O mainly grows along the *a*-axis and the *c*-axis without external interaction. The product morphology is quadrilateral block crystal if the growth rates in the two directions are similar. When methanol is used as the anti-solvent, the hydroxyl group of methanol interacts with the oxygen in the CaSO_4_·2H_2_O crystal and is enriched in the *c*-axis direction during the crystallization of CaSO_4_·2H_2_O. However, the CaSO_4_·2H_2_O crystal growth will be subject to a relatively small inhibition effect due to the small volume of methanol molecular side chains, which results in a small difference in the growth rates of the *a*-axis and *c*-axis. Finally, the morphology of Product A appears as butterfly-like lamellar crystals. When n-propanol is used as the anti-solvent, the n-propanol is enriched in the *c*-axis direction of the CaSO_4_·2H_2_O crystal during the growth of the CaSO_4_·2H_2_O crystal, which suppresses the growth of the crystal in the *c*-axis direction. At the same time, the side chain of n-propanol is large, and n-propanol has a significant inhibitory effect on the growth of the CaSO_4_·2H_2_O crystal due to the long chain. Finally, the morphology of Product C appears as a hexagonal prismatic crystal. The difference in the morphology between Products A and C can be attributed to the difference in the inhibition effect of the anti-solvent during CaSO_4_·2H_2_O crystal formation, resulting in different growth rates of the *a*- and *c*-axes. Therefore, if the calcium ion chelating agent is adsorbed in the *a*-axis direction to limit the growth rate in the *a*-axis direction, it will lead to an increase in the precipitation induction time and change the morphology of the product [28].

**Figure 6:**
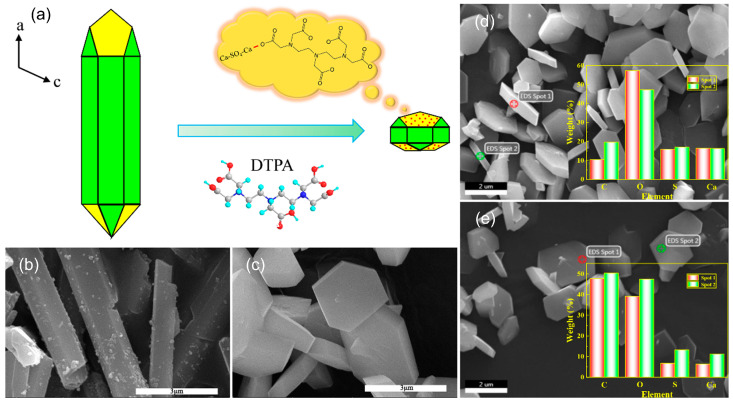
The effect of DTPA on product morphology. (**a**) Schematic diagram of the effect of DTPA on product morphology. (**b**) Morphology of the product without the addition of DTPA. (**c**) Morphology of the product after adding DTPA. (**d**) Side EDS image of the product after adding DTPA and (**e**) EDS image of the end face of the product after adding DTPA.

Diethylenetriaminepentaacetic acid (DTPA) (Figure S4), which is an effective calcium chelator, is selected to control the growth rate in the *a*-axis direction. DTPA was first dissolved in a phosphogypsum solution and chelated with calcium ions of CaSO_4_·2H_2_O. Subsequently, n-propanol is used as an anti-solvent to precipitate CaSO_4_·2H_2_O crystal (Product D). During the growth process, the DTPA will be enriched in the *a*-axis direction of the CaSO_4_·2H_2_O crystal along with the calcium ions (Figure 6a). The *a*-axis of the crystal can inhibit the growth of the *a*-axis direction and reduce the length of the product due to the large molecular size of DTPA. Figure 6b,c shows the morphologies of the product before and after the addition of DTPA, respectively. When DTPA is not added, the product morphology is a hexagonal prism-like crystal. The product morphology becomes a hexagonal prism-thin crystal after adding DTPA, which is consistent with the theoretical prediction. From the structure of DTPA, it can be seen that DTPA contains a large number of carbon elements, and the enrichment of DTPA on the surface of Product D will inevitably cause a change in the element content on the surface of the product. The element content of the side and end faces of Product D was characterized by the energy spectrum, as shown in Figure 6d,e. The carbon content of the end face and side in Product D is about 44.44% and 15.00%, respectively, which reveals that DTPA chelated with the calcium ion of the *a*-axis direction. The result further confirms that organic molecules adhered to the surface of CaSO_4_·2H_2_O crystals will affect the growth rate of the product in different directions and further change their morphology. 

**Figure 7:**
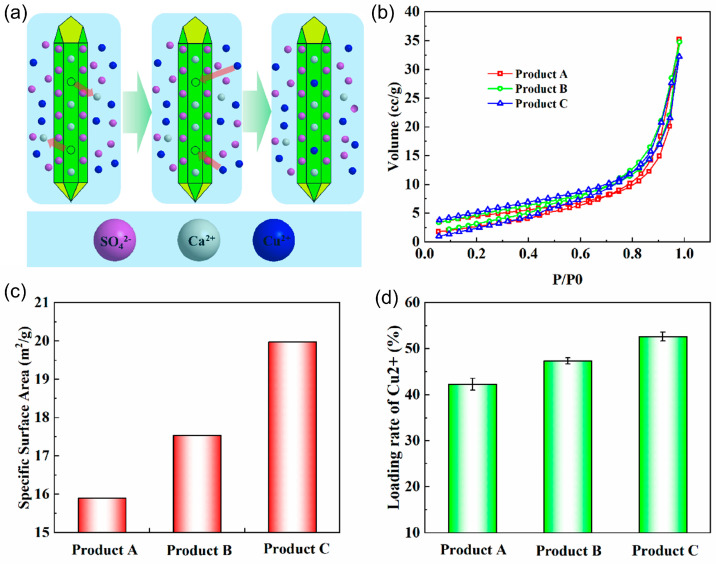
(**a**) Cu^2+^ attached schematic diagram. (**b**) Nitrogen adsorption-desorption isotherms of the product. (**c**) Product surface area and (**d**) Cu^2+^ loading rate graph.

Compared with phosphogypsum, the obtained CaSO_4_·2H_2_O product has no obvious toxicity to cells and no irritation to normal skin by the cell and animal experiments, indicating that the product can be effective in the field of biomedicine (Figures S5 and S6, Supplementary Materials). Since Cu^2+^ has excellent antibacterial properties, the Cu^2+^ solution is used to load Cu^2+^ in the product to expand its application field. The loading process mainly has two stages, as shown in Figure 7a. First, the ${\mathrm{SO}}_{4}^{2-}$ and Ca^2+^ entered the solution after the dissolution of the CaSO_4_·2H_2_O crystal on the surface product, and then a part of the Cu^2+^ in the solution adhered to the surface of the product. This process was equivalent to the replacement of Ca^2+^ with Cu^2+^ on the product surface [16]. Therefore, the specific surface area of the product has an important influence on the loading efficiency of the product Cu^2+^. Figure 7b is the adsorption and desorption curve of each product N_2_. The specific surface area of each product is calculated by the BET algorithm as shown in Figure 7c, the specific surface areas of products A, B, and C are 15.90 m^2^/g, 17.54 m^2^/g, and 19.98 m^2^/g, respectively. Figure 7d shows the Cu^2+^ loading efficiency of the product, where the Cu^2+^ loading efficiency of Products A, B, and C are 42.27%, 47.36%, and 52.67%, respectively. Product C with hexagonal prismatic crystal morphology has the largest specific surface area, and Product C also has the highest Cu^2+^ loading efficiency. 

## 3. Materials and Methods

### 3.1. Materials

Phosphogypsum, from Wengfu Group, Guiyang, Guizhou Province, China. Methanol (content, ≥99.0%, Tianjin Fuyu Fine Chemical Co., Ltd., Tianjin, China), ethanol (content, ≥99.0%, Tianjin Fuyu Fine Chemical Co., Ltd.), n-propanol (content, ≥99.0%, Tianjin Fuyu Fine Chemical Co., Ltd.), and CuSO_4_·5H_2_O (content, ≥99.0%, Tianjin Zhiyuan Chemical Reagent Co., Ltd., Tianjin, China).

### 3.2. Synthesis of CaSO_4_·2H_2_O Crystal

**Figure 8:**
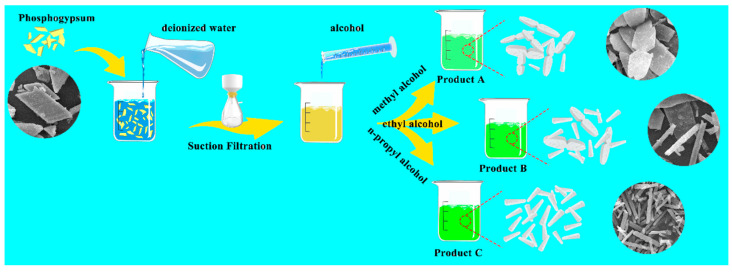
Product preparation experiment flow chart.

The product preparation process is shown in Figure 8. A total of 20 g of phosphogypsum was placed in a beaker, and 4000 mL of deionized water was added. The beaker was then placed on a magnetic stirrer with a rotating speed of 800 r/min and stirred at room temperature for 2 h, and then the insoluble matter was removed by filtering to obtain a transparent phosphogypsum solution. Finally, the 200 mL of phosphogypsum solution and 60 mL of alcoholic solvents were respectively heated to 55 °C, and then the alcohol was slowly added to the phosphogypsum solution to form target products (different morphology CaSO_4_·2H_2_O crystals). The products prepared with methanol, ethanol, and n-propanol as the anti-solvent are denoted as Product A, Product B, and Product C, respectively. To further assess the effects of the mixing ratio of anti-solvent on product morphology, 40, 80, and 100 mL of n-propanol were added to the 200 mL of phosphogypsum solution to obtain products C1, C2, and C3, respectively.

### 3.3. Characterization

The morphology of the product was observed by scanning electron microscopy (SEM) (Quanta FEG 250, FEI, Hillsboro, OR, USA). The elemental composition of the product and the binding energy of surface feature element modification were analyzed by X-ray photoelectron spectroscopy (XPS) (KAlpha+, Thermo Scientific, Waltham, MA, USA) changes to determine whether the product interacts with the surface during crystallization. The composition of the powder product was determined by powder X-ray diffraction (PXRD) (Bruker AXS D8 Advance, Billerica, MA, USA). The thermal stability of the product was determined by thermogravimetric analysis (TGA) (TGA55, TA, Newark, DE, USA) and differential scanning calorimetry (DSC) under nitrogen protection. Fourier transform infrared spectroscopy (FTIR) (Nicolet iS50, TA, Waltham, MA, USA) was used to determine product functional groups. The specific surface area of the products was analyzed using the Brunauer–Emmett–Taylor (BET) method (NOVA 1000e, Quantachrome, Boca Raton, FL, USA). The Cu(II) concentrations were determined by atomic absorption spectrophotometry (AAS) (240FS AA, Agilent, Santa Clara, CA, USA).

### 3.4. Loading Experiment

In this experiment, CuSO_4_·5H_2_O is selected as the loading agent to load copper ions. A total of 1g of the sample (Products A, B, and C) was added to 50 mL of CuSO_4_·5H_2_O solution at a concentration of 10mg/L (*C*_0_). Then the mixture is placed in a water bath constant temperature oscillator (the temperature is 25 °C, and the oscillation frequency is 180 r/min). After the reaction of 6h, the samples loaded with the copper ions are obtained by filtration and drying. In addition, the remaining CuSO_4_·5H_2_O solution is measured again for concentration, and the value is recorded as *C_t_*. The loading efficiency (*R*) is calculated by Equation (3):

(3) $$\begin{array}{c} R=\frac{\left(C_{0}-C_{t}\right)}{C_{0}}\times 100\% \end{array}$$

## 4. Conclusions

In summary, the anti-solvent method was used to directly purify CaSO_4_·2H_2_O from phosphogypsum. The experimental operation is simple, the reaction conditions are mild, the morphology control is excellent, and the energy consumption is low. During the growth of the CaSO_4_·2H_2_O crystal, the experimental results show that the antisolvent interacts with oxygen in the crystals and adheres to the *c*-axis direction of crystals, which inhibits the growth rate on the *c*-axis direction. The inhibiting effect will become stronger as the length of the anti-solvent chain increases, which results in the growth rate of crystal on the *a*-axis being significantly higher than that of the *c*-axis, and the morphology of the product changing from butterfly-like flake crystals to hexagonal prism-like crystals. Further, the DTPA was used to chelate with Ca^2+^, which greatly reduced the growth rate on the *a*-axis of CaSO_4_–2H_2_O crystals, and the morphology of the product was transformed from hexagonal prismatic to hexagonal prismatic thin crystal. In addition, compared to Products A and B, Product C exhibits the highest specific surface area. Consequently, Product C demonstrates a high Cu^2+^ loading efficiency (52.67%, 19.98 m^2^/g), suggesting that Product C holds greater potential for application in biomedical fields (copper ions possess antimicrobial properties). This work provides technologies for the fine utilization of phosphogypsum, which will greatly promote the resourceful and environmentally friendly utilization of phosphogypsum.

## Data Availability

The original contributions presented in the study are included in the article, further inquiries can be directed to the corresponding author.

## Supplementary Materials

The following supporting information can be downloaded at: https://www.mdpi.com/article/10.3390/molecules29163910/s1, Figure S1. (a) Photo of phosphogypsum after drying, (b) SEM image of phosphogypsum, (c) XRD pattern of phosphogypsum, and (d) XPS curve of phosphogypsum; Figure S2. SEM micrographs of Products C1 (left), C2 (middle), and C3 (right), acquired at varying mixing ratios of anti-solvent (2:10, 4:10, and 5:10); Figure S3. The magnified PXRD patterns of product and Standard XRD pattern of CaSO_4_·2H_2_O; Figure S4. The molecular structure of DTPA; Figure S5. The effect of Product C and phosphogypsum on the proliferation of mouse macrophages. (Note: The above figures (a) and (b) correspond to Product C and phosphogypsum in sequence. Compared with the blank group *** P < 0.001); Figure S6. Dermal toxicity tests: (a) phosphogypsum and (b) product C; Table S1. Approximate atomic percent content of three main elements Ca, O, and S in the product; Table S2. Characteristic peaks of the O element.
