# Supplementary material for: High-Efficiency Purification and Morphology Regulation of CaSO4·2H2O Crystals from Phosphogypsum
Source: Molecules. 2024 Aug 19;29(16):3910. doi: 10.3390/molecules29163910 (PMC11357588; doi:10.3390/molecules29163910)
Supplement: Supplementary file 1 [file molecules-29-03910-s001.zip › molecules-3119155-supplementary.pdf]

Support information

Supplementary Note 1 (Cell experiment):

Purpose of the experiment: Using the MTT method to detect the activity of drugs on cell proliferation.

Experiment equipment: CO_2 incubator, inverted microscope, ultra-clean workbench, microplate reader, 96-well cell culture plate, cell culture flask, ultrapure water preparation instrument, automatic high-pressure steam sterilizer.

Experimental reagents: 3-(4,5-DIMETHYL-2-THIAZOL-2-YL)-2,5-DIPHENYL-2H-TETRAZOLIUM BROMIDE (MTT), Dimethyl sulfoxide(DMSO), DMEM medium, trypsin, Fetal bovine serum, Calf serum, Penicillin/streptomycin mixture (P/S).

Configuration of main reagents: (1) PBS: NaCl 8.0g, KCl 0.2g, Na_2HPO_4-$12H_2O$ 29g, KH_2PO_4 0.2g, in 800 ml ultrapure water, Adjust pH to 7.4, Constant volume of 100 ml, high-pressure steam sterilization. (2) Preparation of MTT: Weigh 0.5 g of MTT, dissolve it in 100 ml of phosphate-buffered saline (PBS) to obtain an MTT solution with a concentration of 5 mg/ml, filter it with a 0.22 μm filter to remove bacteria in the solution, and store at 4°C in the dark.

Experimental methods: The MTT method was used to detect the effect of monomeric compounds on the proliferation activity of mouse macrophage RAW264.7, and to screen safe and effective drug concentrations. Observe under an inverted microscope, take the cells in the logarithmic growth phase, adjust the cell concentration to 2×104/mL and inoculate in a 96-well plate, 100μL per well, and incubate at 37°C, 5% CO_2 incubator for 24 hours until the cells adhere to the wall. , adding different molar concentrations of medicinal liquid as the administration group, setting the concentration to 6.25, 12.5, 25, 50, and 100 μmol/L. A blank control (only cell culture medium was added) was set up, and the culture was continued for 24 hours. 20 μL of MTT (5 mg/mL PBS

solution) was added to each well, and the culture was continued for 4 hours. After the culture, the supernatant was aspirated, 150 mL of μL of DMSO was shaken in the dark for 10 min, and the absorbance was detected at 570 nm.

Experimental results: Compared with the blank control group, the cell viability of Product C at 100 $\mu g \cdot mL^{-1}$ was greater than 90%, and there was no obvious toxicity to mouse macrophage RAW264.7 cells; The viability was 47.68%. At 12.5 $\mu g \cdot mL^{-1}$, the cell viability was 81.42%. Phosphogypsum had certain toxicity to mouse macrophage RAW264.7 cells. The results of Product C and phosphogypsum are shown in Fig. S5.

Supplementary Note 2 (Animal experiment)

Purpose of the experiment: To observe the skin toxicity of phosphogypsum and the extracted Product C, and to evaluate its drug safety.

Experiment equipment: Eight New Zealand white rabbits, half male and half male, weighing 2.5-3.0 kg, were provided by the Key Laboratory of Natural Product Chemistry, Chinese Academy of Sciences, Guizhou Province, and the experimental animal production license number: SCXK 2015-0001. They were reared in an environment with a room temperature of 20-24°C and relative humidity of 40%-70%, circadian rhythm for 12 h, free drinking water, and solid feed. This experimental study was approved by the Laboratory Animal Ethics Committee of Guizhou Medical University and complies with the requirements of the general guidelines for animal ethics review in Guizhou Province.

Experimental method: Take 8 New Zealand big-eared white rabbits, half male, and half male, and 24 hours before administration, both sides of the spine of the rabbits were dehaired, and the depilation range on each side was 7 cm × 7 cm. damage. Grouping and administration: 8 New Zealand white rabbits were divided into 2 groups by random number table method, with 4 rabbits in each group. Using the method of comparing the left and right sides of the same body, the skin of the left hair removal area of the two groups of rabbits was evenly sprayed with phosphogypsum and

a saturated aqueous solution of Product C, twice a day, for 14 consecutive days, and the right amount of water was applied as a blank control. Observation indicators: Under natural light, visually observe whether there is erythema, edema, etc. on the skin of the application site.

Experimental results: There was no erythema and edema in the Product C group. The left and right skin irritation response scores in the phosphogypsum group were 0 at each time point, and there was no significant difference between the left and right sides ($P>0.05$). Indicates that a single dose of Product C is non-irritating to normal skin. On the second day of the phosphogypsum group, the left skin irritation response score was 1 point at the 24-hour time point, and barely erythema appeared, which disappeared after the third day. It shows that the single administration of phosphogypsum has certain irritation to normal skin (Fig. S6).

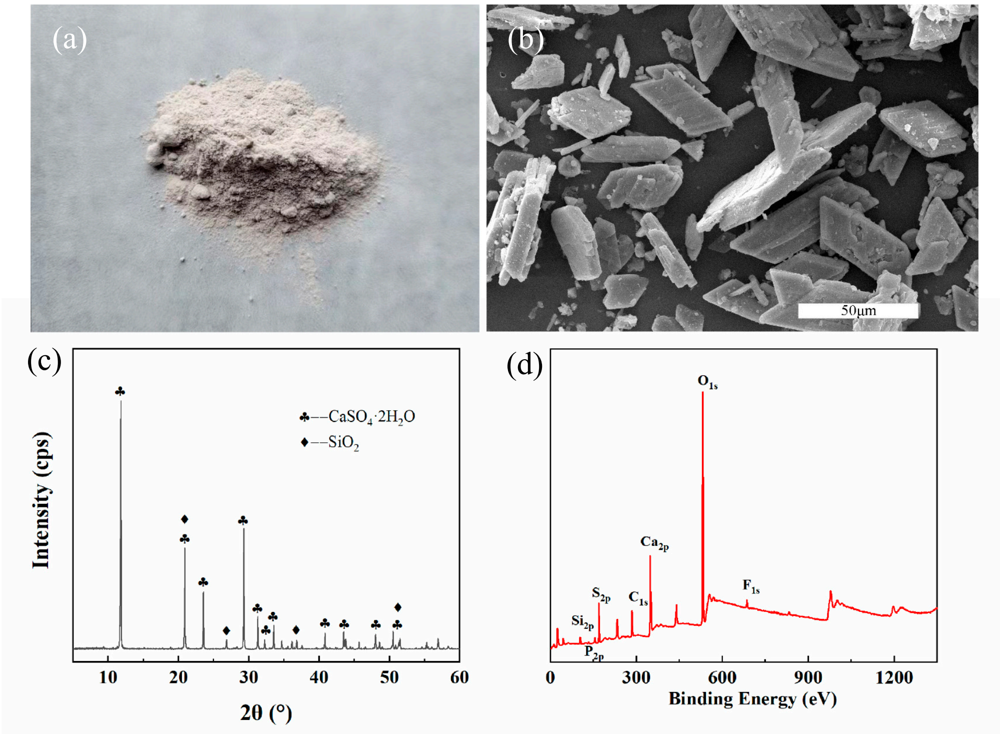

Fig. S1. (a) Photo of phosphogypsum after drying, (b) SEM image of phosphogypsum, (c) XRD pattern of phosphogypsum, and (d) XPS curve of phosphogypsum

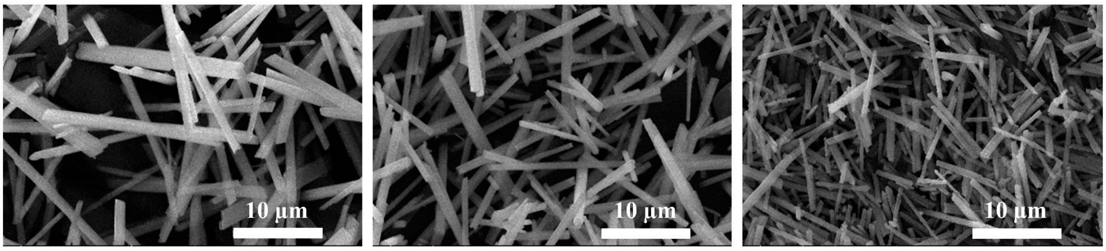

Fig. S2. SEM micrographs of Products C1 (left), C2 (middle), and C3 (right), acquired at varying mixing ratios of anti-solvent (2:10, 4:10, and 5:10).

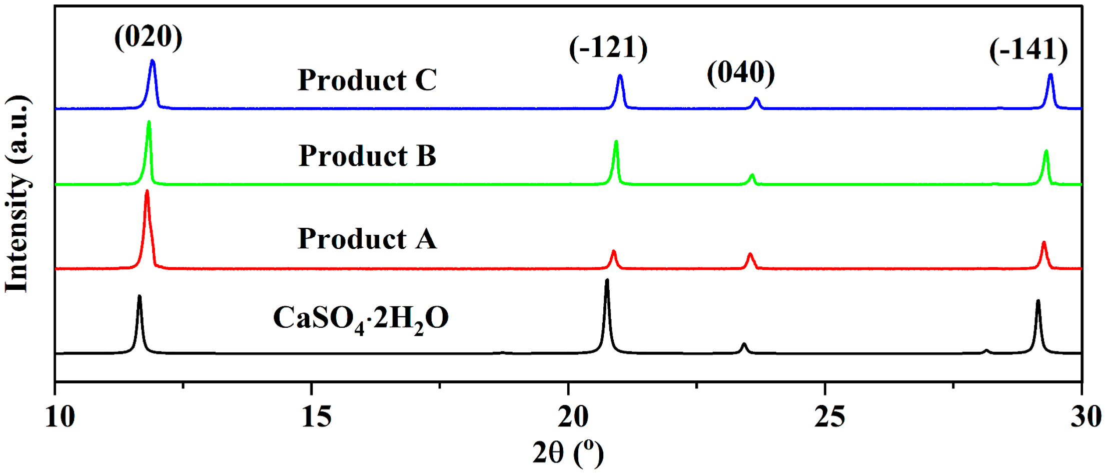

Fig. S3. The magnified PXRD patterns of product and Standard XRD pattern of $CaSO_4 \cdot 2H_2O$.

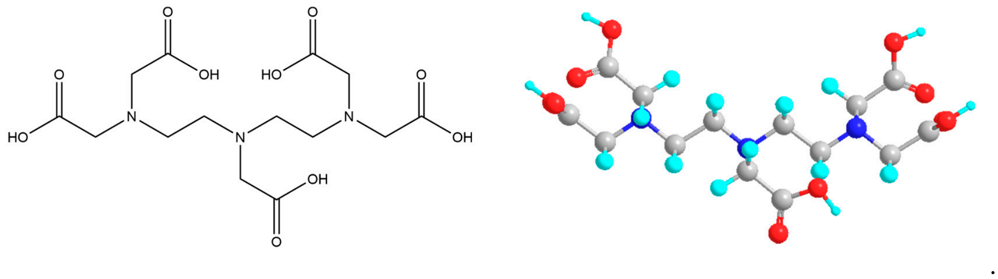

Fig. S4. The molecular structure of DTPA

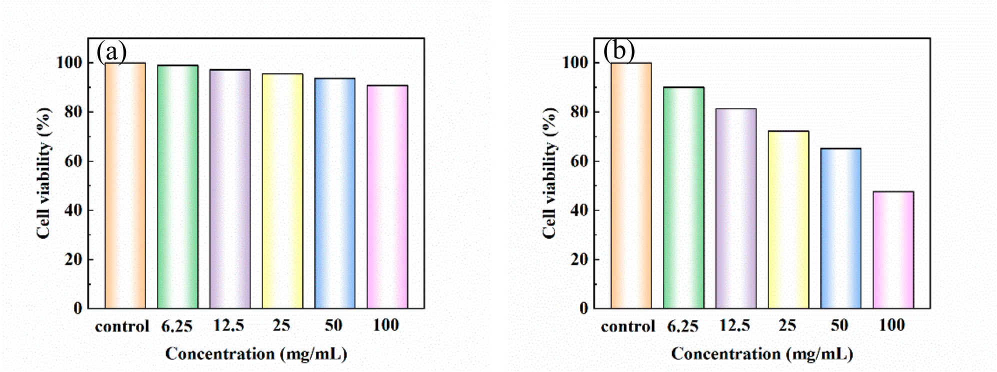

Fig. S5. The effect of Product C and phosphogypsum on the proliferation of mouse macrophages. (Note: The above figures (a) and (b) correspond to Product C and phosphogypsum in sequence. Compared with the blank group ***P<0.001)

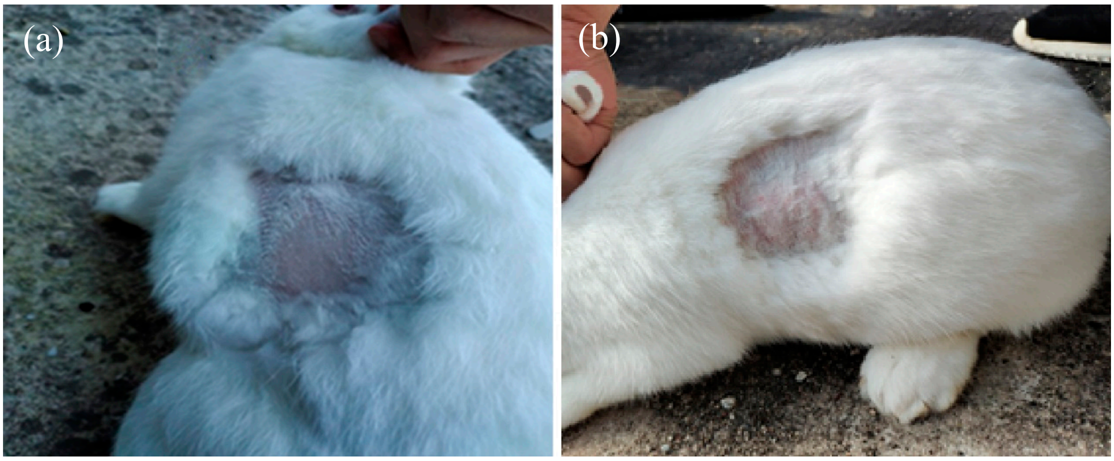

Fig. S6. Dermal toxicity tests: (a) phosphogypsum and (b) product C

Table S1 Approximate atomic percent content of three main elements Ca, O, and S in the product

Element	Atomic (%)		
	Product A	Product B	Product C
Ca	13.82	16.24	16.05
O	58.75	54.65	55.13
S	13.01	14.75	14.68

Table S2. Characteristic peaks of the O element

	Oxygen characteristic peaks	
	O-S	H-O-H
Product A	532.39	533.88
Product B	532.15	533.74
Product C	532.10	533.65
